# Costs of an Alcohol Measurement Intervention in Three Latin American Countries

**DOI:** 10.3390/ijerph19020700

**Published:** 2022-01-08

**Authors:** Adriana Solovei, Jakob Manthey, Peter Anderson, Liesbeth Mercken, Eva Jané Llopis, Guillermina Natera Rey, Augusto Pérez Gómez, Juliana Mejía Trujillo, Inés Bustamante, Marina Piazza, Alejandra Pérez de León, Miriam Arroyo, Hein de Vries, Jürgen Rehm, Silvia Evers

**Affiliations:** 1 Department of Health Promotion, CAPHRI Care and Public Health Research Institute, Maastricht University, P. Debyeplein 1, 6229HA Maastricht, The Netherlands; peteranderson.mail@gmail.com (P.A.); liesbeth.mercken@ou.nl (L.M.); eva.jane@esade.edu (E.J.L.); hein.devries@maastrichtuniversity.nl (H.d.V.); 2 Institute for Clinical Psychology and Psychotherapy, TU Dresden, Chemnitzer Str. 46, 01187 Dresden, Germany; jakob.manthey@tu-dresden.de (J.M.); jtrehm@gmail.com (J.R.); 3 Center for Interdisciplinary Addiction Research (ZIS), Department of Psychiatry and Psychotherapy, University Medical Center Hamburg-Eppendorf, Martinistr. 52, 20246 Hamburg, Germany; 4 Department of Psychiatry, Medical Faculty, University of Leipzig, Semmelweisstraße 10, 04103 Leipzig, Germany; 5 Population Health Sciences Institute, Newcastle University, Baddiley-Clark Building, Richardson Road, Newcastle upon Tyne NE2 4AX, UK; 6 Department of Health Psychology, Faculty of Psychology and Educational Sciences, Open University Postbus 2960, 6401DL Heerlen, The Netherlands; 7 ESADE Business School, University Ramon Llull, Av. de Pedralbes, 60, 62, 08034 Barcelona, Spain; 8 Institute for Mental Health Policy Research, CAMH, 33 Russell Street, Toronto, ON M5S 2S1, Canada; 9 Instituto Nacional de Psiquiatría Ramón de la Fuente Muñiz, Calz México-Xochimilco 101, Huipulco, Mexico City 14370, Mexico; naterar@imp.edu.mx (G.N.R.); apdeleon@imp.edu.mx (A.P.d.L.); mirabel@gmail.com (M.A.); 10 Corporación Nuevos Rumbos, Calle 108 A # 4-15, Bogota 110111, Colombia; aperez@nuevosrumbos.org (A.P.G.); jmejia@nuevosrumbos.org (J.M.T.); 11 School of Public Health and Administration, Universidad Peruana Cayetano Heredia, Av. Honorio Delgado 430, Urb Ingeniería, Lima 15102, Peru; ines.bustamante@upch.pe (I.B.); marina.piazza@upch.pe (M.P.); 12 Dalla Lana School of Public Health, University of Toronto, 155 College Street, 6th Floor, Toronto, ON M5T 3M7, Canada; 13 Department of Psychiatry, University of Toronto, 250 College Street, 8th Floor, Toronto, ON M5T 1R8, Canada; 14 Department of International Health Projects, Institute for Leadership and Health Management, I.M. Sechenov First Moscow State Medical University, Trubetskaya Str., 8, b. 2, 119992 Moscow, Russia; 15 Department of Health Services Research CAPHRI Care, Public Health Research Institute, Maastricht University, POB 616, 6200MD Maastricht, The Netherlands; s.evers@maastrichtuniversity.nl

**Keywords:** alcohol measurement, alcohol control, costs, training, community support

## Abstract

Alcohol measurement in health care settings is an effective intervention for reducing alcohol-related harm. However, in many countries, costs related to alcohol measurement have not yet been transparently assessed, which may hinder its adoption and implementation. Costs of an alcohol measurement programme in three upper-middle-income Latin American countries were assessed via questionnaires and compared, as part of the quasi-experimental SCALA study. Additional to the intervention costs, the costs of three implementation strategies: standard training and clinical package, intensive training and clinical package, and community support, were assessed and subsequently translated into costs per additional alcohol measurement session. Results demonstrated that costs for one alcohol measurement session ranged between Int$ 0.67 and Int$ 1.23 in Colombia, Int$ 1.19 and Int$ 2.57 in Mexico, and Int$ 1.11 and Int$ 2.14 in Peru. Costs were mainly driven by the salaries of the health professionals. Implementation strategies costs per additional alcohol measurement session ranged between Int$ 1.24 and Int$ 6.17. In all three countries, standard training and a clinical package may be a promising implementation strategy with a relatively low cost per additional alcohol measurement session.

## 1. Introduction

Alcohol use is one of the leading preventable risk factors for health and social harms, causing an estimated three million deaths worldwide each year [1]. More than 200 disease and injury categories are either partly (e.g., various cancer subtypes, ischemic heart disease, liver cirrhosis, and traffic injury) or entirely (e.g., alcohol-use disorders and foetal alcohol syndrome) caused by alcohol [2]. This includes negative social consequences, which go beyond the health care sector, such as interpersonal violence, self-harm, vandalism, criminality, and work-related losses of productivity [3]. A recent review [4] found that the costs associated with alcohol constitute around 2.6% of the GDP (95% CI: 2.0% to 3.1%) in middle- and high-income countries, including health care and criminal justice costs, as well as losses in productivity. Latin America is a region with a relatively high magnitude of alcohol-attributable disease burden, with around 6% of the deaths and 6% of DALY’s in the region caused by alcohol [2]. In the three countries addressed in this paper, alcohol consumption is a top-five leading cause of mortality and premature death [5]. Manthey and colleagues [6]—before the COVID-19 pandemic—estimated an increase in overall alcohol consumption in the region from 2018 to 2030. Therefore, it is crucial to implement interventions and policies to prevent and manage alcohol-related harm from a public health perspective.

The World Health Organization’s (WHO) SAFER alcohol control initiative entails five cost-effective strategies to combat alcohol-related harm [7]. One of these strategies is facilitating population-level health service access to the measurement of alcohol consumption, and delivering brief advice and treatment to individuals identified as at risk. Measurement of alcohol consumption (henceforth: alcohol measurement) is the assessment of a patient’s alcohol consumption by a health care provider (henceforth: provider), typically using a standard questionnaire, for example, AUDIT-C [8]. The alcohol measurement can be either positive, meaning that the patient scores above a certain predetermined threshold for hazardous drinking, or negative, i.e., the patient scores under the respective threshold. As recommended in several guidelines on this matter [9,10], patients with positive alcohol measurements should receive brief advice from the provider immediately after the alcohol measurement session. This is a time-limited effort in which the health care professional provides information and advice aimed at increasing the patient’s motivation to avoid or reduce alcohol use, thus reducing the negative health consequences associated with it [11]. Patients at risk may also receive a referral to treatment, such as to an inpatient/outpatient treatment or supportive services if the patient shows clear signs of (mental) health problems caused by his/her alcohol use.

Substantial evidence indicates that alcohol measurement in health care settings is an (cost-) effective strategy to prevent and manage alcohol-related health harm [12,13]. However, in many regions of the world, this intervention is not yet widely adopted as routine practice [14]. One key barrier to its widespread implementation refers to the lack of insights regarding how costly the implementation of such a programme may be [15]. In settings of more limited resources, detailed assessments of the costs needed to implement a health programme may be of particular importance. Moreover, a detailed and transparent cost assessment may be relevant in the budgeting process of public health policies or in decisions regarding cutting certain cost components in order to save costs [16]. In addition, some cost components may be irrelevant in certain contexts (e.g., printing costs that are not needed in a web-based intervention). Additionally, a transparent cost assessment can be used as a basis for economic evaluations of the intervention in different settings, potentially demonstrating whether the intervention may result in cost-effective health gains (e.g., through decreased mortality and avoided loss of productivity). 

Only a few studies have estimated the costs of alcohol measurement programmes, with some substantial differences among various contexts [11,17]. A review by Bray and colleagues [17] reported costs of alcohol measurement sessions varying as much as between USD 0.51 [18] and USD 93 [19]. These substantial variations were largely driven by the time spent on each session, the complexity of the intervention and the country where it is implemented. The scarcity and heterogeneity of existing evidence in this field stress the need for detailed and transparent cost assessments of alcohol measurement interventions in various regions of the world. Moreover, programme costs related to different implementation strategies (e.g., provision of training or community support) are also often less transparently assessed [20]. 

Based on this, the current paper aims to provide an assessment and comparison of the following costs in three upper-middle-income Latin American countries, namely Colombia, Mexico, and Peru: (1) consultation costs of one alcohol measurement session in primary health care (PHC) settings; and (2) programme costs of three implementation strategies (specified in the next section), including the costs per additional alcohol measurement session.

## 2. Materials and Methods

### 2.1. Study Design

**Figure 1:**
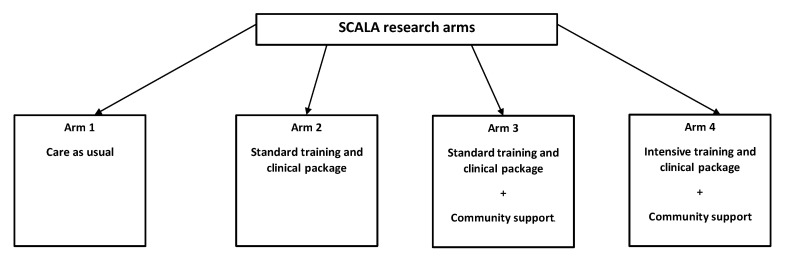
Implementation strategies in the SCALA research arms.

The costs associated with the set-up and implementation of an alcohol measurement programme were collected as part of the “Scale-up of Prevention and Management of Alcohol Use Disorders and Comorbid Depression in Latin America” (SCALA) study [21]. SCALA is a quasi-experimental implementation science study, which seeks to upscale the delivery of alcohol measurement at the municipal level in Colombia, Mexico, and Peru, through a multi-component approach (see Figure 1). Specifically, the following implementation strategies are included in the SCALA study: (1) provision of standard training combined with standard clinical package, e.g., clinical pathway and support materials (henceforth: standard training and clinical package); (2) provision of a more intensive training combined with a longer clinical package (henceforth: intensive training and clinical package); and (3) provision of community support. The strategies were compared to a control group, i.e., care as usual [22,23]. In each of the three participating countries, two municipalities were recruited (one without community support, and one with community support, see Table A1 for more information), each with 9–10 participating PHC centres (PHCC). This resulted in 58 participating PHCCs, spread over four study arms, using clustered randomisation. The implementation phase was planned to last 18 months and started in September–October 2019; however, it was paused mid-March 2020, due to the COVID-19 pandemic, which disrupted the PHC services in the three countries. Data included for this analysis relates, therefore, to the first five implementation months of SCALA. 

### 2.2. Implementation Strategies

The implementation strategies (see Table A2) were executed as follows: participants in arm 1 (care as usual) received a booklet describing a pathway for delivering alcohol measurement and subsequent interventions and paper tally sheets with the AUDIT-C questionnaire (three items), which providers could use to deliver the intervention (for more information, see www.scalaproject.eu, accessed on 25 October 2021). No other support materials and activities were offered. Participants in arm 2 received training (consisting of one session) before the implementation, in which they were trained to deliver alcohol measurement and subsequent interventions using the same pathway as in arm 1. Additionally, a booster session of one hour was planned to be given to providers; however, this could not be implemented in all PHCCs, due to the COVID-19 lockdown, and was therefore not included in the current costs assessment. 

PHCCs in arm 3 (intervention municipality) received the same training and clinical package materials as in arm 2, and community support aimed at helping the adoption and implementation of the intervention. The community support consisted, among others, of regular performance feedback and support given by the project team to the providers during every implementation month (see Table A3). Community support also included (indirect) support from a community advisory board (CAB). The CAB was created in each intervention municipality, consisting of 10–12 relevant stakeholders for the public health domain, and met two times in Peru and three times in Colombia and Mexico throughout the set-up and implementation phase of the project [24]. 

In arm 4 (intervention municipality), PHCCs received the same community support as in arm 3, along with more intensive training and clinical package than arms 2 and 3. The more intensive training consisted of one additional training session of 2 hours in Mexico and Peru, and in Colombia of 30 additional minutes added to the main session, compared to the standard training (arm 3). The more intensive clinical package consisted of administrating the full AUDIT questionnaire (10 items) during the alcohol measurement sessions rather than AUDIT-C (for more information, see www.scalaproject.eu, accessed on 25 October 2021). 

### 2.3. Costs Identification, Measurement, and Valuation

**Figure 2:**
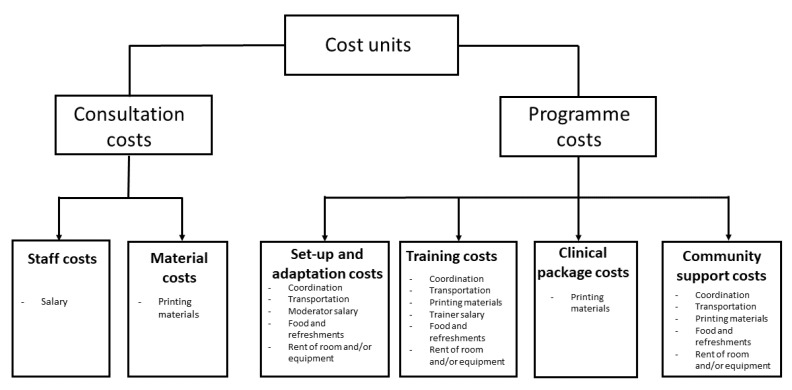
Cost units identified, measured, and valued in the SCALA project.

In a cost analysis it is important to identify, measure, and value activities. For this study, cost units were identified from existing literature and based on discussions with the local research teams and/or local PHC managers (see Figure 2). The final list of identified cost units is operationalised in Table 1 and Table A3 and is explained later in this paper. A health care system perspective was used, meaning that the costs related to the implementation of the intervention were assessed (i.e., costs of the resources used in the set-up and delivery of brief alcohol advice) rather than the full societal cost of the intervention. Research-related costs, such as the time needed by the research team to explain the procedures of the study and to recruit PHCCs, or the time needed by providers to fill in questionnaires, were not included in the assessment. 

The measuring of cost units was conducted through three main sources: the local research team, the PHCC managers, and the participating providers. The local research team received a question list to specify the time and costs spent on various activities. The PHCC managers (i.e., *n* = 18 in Mexico, *n* = 20 in Colombia, *n* = 20 in Peru) gave information about the providers’ salaries. Providers (*n* = 53 in Colombia, *n* = 25 in Mexico, *n* = 75 in Peru) gave information about how much time they spent on average delivering alcohol measurement, brief advice, and referral to treatment. For costs valuation, the local prices and costs were converted to International Dollars (Int$), using the purchasing power parity (PPP) exchange rates (1Int$ = 1349.01 COP; 1Int$ = 9.31 MXN; 1Int$ = 1.74 PEN), to allow for easier inter-country comparison of costs [25]. 

### 2.4. Consultation Costs

Consultation costs refer to the direct costs arising from delivering the intervention to a new patient and include staff costs and material costs. Staff costs were calculated by multiplying the average hourly salary of the provider (see Table A4) by the average amount of time used for an alcohol measurement session, brief advice session, and referral to treatment session. The range of providers delivering the intervention in the three countries included mainly GPs and nurses, as well as social workers, psychologists, and other professions. 

First, the number of alcohol measurement sessions delivered by each different profession was assessed, per country, and then these proportions were used to calculate the average staff costs per alcohol measurement session, accounting for variations in staff costs. Material costs were assessed by multiplying the number of pages used for a session with the costs for printing one page. Finally, consultation costs were calculated as the sum of staff costs and material costs, per session.

### 2.5. Programme Costs

Programme costs refer to the costs incurred outside the point of delivery of the intervention to beneficiaries. In SCALA, the programme costs include the set-up and adaptation costs, and the costs of the three implementation strategies that were carried out: standard training and clinical package, intensive training and clinical package, and community support. 

Set-up and adaptation costs refer to costs incurred between the decision to implement the intervention and the start of its delivery (including the delivery of the implementation strategies). The identified set-up costs included coordinating PHCCs’ and providers’ participation in the SCALA intervention. Adaptation costs included the costs of adjusting and tailoring the clinical package materials to the local contexts. In SCALA this was conducted in each country with two user panels: one with a group of 10 health care providers and one with 10 patients. Identified costs included user panel coordination, transportation, food and refreshments, printing materials, moderator salary, technical equipment, and materials adaptation coordination. Research-related costs, e.g., the coordination of data collection and survey completion, were not included in the general start-up and adaptation costs, as these do not apply to the actual implementation of the intervention. 

The costs of the three implementation strategies were identified and assessed as follows. For both (i) standard training and clinical package and (ii) intensive training and clinical package, cost units included coordination of the training, transportation for participants and/or organisers, food and refreshments, training materials, technical equipment, trainer salary, and printing clinical package materials. For community support, the identified cost units include coordination of CAB meetings, food and refreshments, materials, venue rent, transportation, and coordination and implementation of supportive actions. These costs were measured through questionnaires filled in by the three local research teams. 

The costs of coordinating and/or delivering the abovementioned activities (i.e., PHCC participation, user panels, training sessions, CAB meetings, and supportive actions) were assessed by multiplying the average hourly wage of the implementers with the time spent by them in preparing and/or delivering each activity. The costs of printing materials were assessed by multiplying the number of double-sided pages used for a session with the costs for printing one double-sided page. Transportation and venue rent costs were assessed per activity, and food and refreshment costs were assessed per portion. 

### 2.6. Costs per Additional Alcohol Measurement Session

The costs for each implementation strategy were divided by the number of providers participating in the respective study arm, and, subsequently, by the average number of additional alcohol measurement sessions delivered by each provider. The number of additional sessions per implementation strategy was assessed through comparison to the study arm in which the implementation strategy was not implemented, i.e., arm 2 vs. arm 1, for standard training and clinical package; arm 3 vs. arm 2 for community support; and arm 4 vs. arm 3 for intensive training and clinical package.

### 2.7. Costs per 10,000 Alcohol Measurement Sessions in SCALA

Additionally, we estimated the costs for 10,000 alcohol measurement sessions in each SCALA arm, using the three abovementioned implementation strategies. The number of 10,000 alcohol measurement sessions was identified as a relevant cost indicator for potential policy implementation and scale-up. For these estimations, the period (i.e., number of years) that would be needed to achieve this number was calculated per SCALA arm, based on the existing alcohol measurement numbers assessed in the five months of implementation. 

Based on these estimated periods, the number of activities related to each implementation strategy that would have to be implemented was assessed, along with the respective costs (see Table A5). This allowed considering both fixed costs (i.e., which do not depend on the number of delivered consultations) and variable costs (i.e., which change depending on the number of delivered consultations). The programme costs were estimated in each country based on the existing number of recruited PHCCs and participating providers in each study arm, as specified in Table 2. Specifically, for the standard training and intensive training implementation strategies, it was estimated that booster sessions of one hour would be given annually to the participating providers. The cost of a booster session was estimated based on the standard training cost units, correspondingly. For community support, it was estimated that one CAB meeting would be organised annually and that supportive actions would be implemented monthly. Finally, the programme costs, including the set-up costs (corresponding to the number of participating PHCCs in each country and arm) and adaptation costs, were added to the (care as usual) consultation costs of 10,000 alcohol measurements. 

### 2.8. Statistical Testing

The statistical significance of differences between countries (within the same arm) and between arms (within the same country) was tested using confidence intervals for two variables assessed at the provider level: (1) the number of delivered alcohol measurement sessions per provider, and (2) the number of minutes spent on these sessions. Confidence intervals were calculated in SPSS 26 with the function ‘explore’, at a 95% confidence level and were compared. Statistical significance of tested differences was indicated by a lack of overlap of the compared confidence intervals. Differences in unit costs and total costs of the programme cost components (i.e., set-up and adaptation, training, community support) were not tested for significance. These costs were assessed at the country level and did not include sufficient variability to allow for statistical tests. 

## 3. Results

### 3.1. Consultation Costs

In all three countries, providers spent on average between 1.6 and 4.8 min for a standard alcohol measurement session (using AUDIT-C), a brief advice session, and a referral to treatment session. Countries showed differences in the professions of providers who gave the intervention. In Colombia and Mexico, over 60% of the sessions were given by GPs, followed by nurses (in Colombia) and psychologists (in Mexico), whereas in Peru a third of the sessions were given by midwives and a third by psychologists, followed by nurses, nurses technicians, and GPs (each under 5%). The average costs of the three types of sessions (alcohol measurement, alcohol measurement and brief advice, and alcohol measurement and referral to treatment), including the costs of the paper tally sheet used to apply the AUDIT-C questionnaire for the alcohol measurement, were, respectively: Int$ 1.19 (CI: 0.97; 2.54), Int$ 2.57 (CI: 2.09; 4.17), and Int$ 1.84 (CI: 1.51; 3.34) in Colombia; Int$ 0.67 (CI: 0.27; 1.04), Int$ 1.62 (0.58; 2.63), and Int$ 1.23 (0.41; 2.01) in Mexico; and Int$ 1.11 (CI: 1.07; 1.15), Int$ 2.14 (CI: 2.05; 2.24), and Int$ 1.45 (CI: 1.38; 1.52) in Peru (see Table 1). The overlaps in confidence intervals show that the differences are not statistically significant between the three countries.

### 3.2. Programme Costs

Set-up costs (in each country calculated for 15 PHCCs that were not in the control arm) were Int$ 2242.5 in Colombia, Int$ 1711.25 in Mexico, and Int$ 1803.10 in Peru. Adaptation costs of the clinical package materials, including two user panels in each country, were Int$ 1332.15 in Colombia, Int$ 1286.02 in Mexico, and Int$ 1308.45 in Peru (see Table A6 for detailed cost units). As mentioned earlier, these costs were assessed at the country level, and therefore the difference between them could not be tested for statistical significance.

Standard training average costs for one provider were: Int$ 31.70 in Colombia, Int$ 36.15 in Mexico, and Int$ 38.68 in Peru (see Table A6 for detailed cost units). In all three countries, in the five months following the training, providers in arm 2 delivered on average more alcohol measurement sessions per month, namely: 2.65 (CI: 0.61; 4.84) in Colombia, 1.64 (CI: 0.61; 2.53) in Mexico, and 1.37 (CI: 0.60; 1.70) in Peru, as compared to providers arm 1, who received no training. The overlap between the confidence intervals shows that these differences are not statistically significant between the three countries. Including the costs for the clinical package materials used in each alcohol measurement session (i.e., informative leaflets), the average costs of this implementation strategy per additional session were: Int$ 2.68 (CI: 1.61; 10.66) in Colombia, Int$ 4.96 (CI: 3.40; 12.35) in Mexico, and Int$ 6.17 (CI: 5.06; 13.37) in Peru. 

Intensive training average costs for one provider were Int$ 36.47 in Colombia, Int$ 63.01 in Mexico, and Int$ 64.14 in Peru. The substantially lower costs in Colombia are primarily due to the shorter format of the intensive training used there, as mentioned above (i.e., one session in Colombia vs. two sessions in Mexico and Peru, see Table A6). In the next five months following the intensive training, trained providers delivered on average more alcohol measurement sessions per month in Mexico (2.46 more sessions per provider; CI: 1.13; 3.71), while no statistically significant difference was noted in Colombia, which is indicated by the fact that the confidence interval includes zero (0.11 more sessions per provider; CI: −2.62; 5.95). In Peru, providers delivered on average fewer alcohol measurement sessions per month compared to those who received standard training (−1.89 sessions per provider; CI: −3.78; −0.01). Confidence intervals also demonstrate that this difference in direction is statistically significant between Mexico and Peru, but not between the other country pairs. The average costs of this implementation strategy per additional session were Int$ 2.90 (CI: 2.08; 5.77) in Mexico.

The average cost of one CAB meeting was Int$ 717.44 in Colombia, Int$ 833.71 in Mexico, and Int$ 605.68 in Peru. The average cost of one month of supportive actions (including set-up, planning, and implementation) delivered to the participating PHCCs in the intervention municipality (*n* = 9 in Mexico, *n* = 10 in Colombia, *n* = 10 in Peru) was Int$ 358.50 in Colombia, Int$ 205.35 in Mexico, and Int$ 144.25 in Peru. The higher costs in Colombia are primarily due to the larger amount of hours spent to implement the supportive actions (see Table A6). In Colombia, in the five months during which community support was given, providers in the PHCCs in arm 3 that received community support, delivered on average 11.02 more alcohol measurement sessions per provider per month (CI: 4.21; 15.03), compared to arm 2, without community support. There were no statistically significant differences in Mexico (0.88 additional sessions per provider, CI: −0.04; 1.86) and Peru (0.53 additional sessions per provider, CI: −0.35; 1.44). The average costs of community support per additional alcohol measurement session were Int$ 1.24 (CI: 0.91; 3.24) in Colombia. Costs were per 10,000 alcohol measurement sessions in SCALA.

Cost estimations for measuring the alcohol consumption of 10,000 patients in the SCALA research settings are depicted in Table 2. They show that, while the standard training and clinical package implementation strategy (arm 2) was estimated to be the cheapest in all three countries, in Colombia and Mexico the strategy would require a longer period to reach the number of 10,000 alcohol measurements, as compared to the community support strategy. The intensive training and clinical package strategy was estimated to be the most expensive strategy in all three countries, with a longer corresponding implementation period in Colombia and Peru to achieve 10,000 alcohol measurements (compared to the other two strategies). In Mexico, the intensive training and clinical package strategy was estimated to lead to 10,000 alcohol measurements in a shorter time than the other two strategies.

## 4. Discussion

This paper aimed to assess the consultation costs of delivering alcohol measurement sessions in PHC settings in three Latin American countries, along with the programme costs of three implementation strategies aiming to support the implementation of this intervention: (1) standard training and clinical package, (2) intensive training and clinical package, and (3) community support, including the costs of these implementation strategies per additional alcohol measurement session. 

Results indicate that one of the main factors determining the consultation costs is the profession of the providers delivering the intervention. Specifically, when the intervention was largely delivered by GP’s the average salaries were higher than when the intervention was delivered more often by nurses or social workers. Another factor determining the costs is the time spent for certain components of the intervention, such as providing an alcohol measurement session, as also demonstrated in previous studies [11,17]. For a more accurate estimation of consultation costs in international settings, it is therefore important to take into account the type and proportions of PHC professionals who would take up the delivery of alcohol measurement, along with the amount of time such sessions would last. Moreover, it is also important to consider that not only the costs, but also the overall uptake, implementation, and effectiveness of the intervention may largely depend on the type of professional who delivers it [26]. 

Regarding the implementation strategies assessed in the current study, some differences in unit costs in the three countries were noticed. For example, the costs of CAB meetings in Mexico were higher than in Colombia and Peru, e.g., due to larger transportation and materials costs. In addition, noticeably, the community support intervention in Colombia resulted in a higher number of additional alcohol measurement sessions, and, correspondingly, in a lower cost per additional alcohol measurement session. This may be due to a more intensive collaboration with the providers who received community support in Colombia. For example, specific barriers encountered by providers in Colombia were tackled more effectively with community support activities, e.g., through creating video tutorials responding to the specific needs of the providers (for more information, see www.scalaproject.eu, accessed on 25 October 2021). Due to the premature stop in data collection as a result of the COVID-19 pandemic, which started in all three countries by end of implementation month 5, we could not assess in more detail the further implementation of the community support. We expect community support activities to have a cumulative effect over months of implementation and in SCALA, the community support activities were designed to be implemented for a period of 18 months, prior to the COVID-19 pandemic. Thus, it could be that the stronger effects of community support in Mexico and Peru would have manifested only after the observed 5 months period, and as such, the costs per additional alcohol measurement session would be smaller than estimated in this paper. Future studies could benefit from collecting data throughout a longer implementation of community support. In addition to impeded program implementation and health care delivery, the COVID-19 pandemic has also been linked to reduced alcohol consumption [27]. During this public health emergency, resources have been prioritised for more pressing health care issues than for preventive measures. In light of the scarcity of health care resources and economic losses, the implementation of routine alcohol measurement in PHC practice seems to be of even lower importance than before the pandemic. Standard training and clinical package had relatively similar costs in the three countries and led to more alcohol measurement sessions, compared to care as usual, in all three countries. Intensive training and clinical package, on the other hand, was substantially cheaper in Colombia, as compared to Mexico and Peru, due to the shorter training format implemented in the country. As the intensive training and clinical package is a more expensive implementation strategy, compared to the standard training and clinical package (due to larger consultation and programme costs), it is, therefore, recommended to carefully consider the type and intensity of training as implementation strategies, based on needs and preferences in local contexts. 

A strength of this study is that it is based on an implemented science approach, which allows realistic costs to be taken into account. The analyses are based on real-life data, adding confidence in their validity and allowing the costs to be assessed per additional patients, whose alcohol use is measured in the different implementation strategies. Moreover, by transparently presenting the main cost categories and showing a comparison between three countries, the study can be used as a basis for budgeting costs of similar interventions in other countries, by adjusting the cost units according to the national/local contexts. 

A limitation of this study is that the effects of the tested implementation strategies may need a longer time to unfold than it was possible to assess in our research. Moreover, possibly, alcohol interventions can have negative short-term impact, e.g., creating productivity losses (e.g., patients take more time off as a result of referrals), which may only pay-off in the long run. Future research may broaden perspectives and include all societal costs relevant to alcohol consumption (for an overview of all relevant costs categories, see [28]). In addition, the estimated costs (e.g., salaries) are based on the implemented SCALA project and may, therefore, vary within the three countries at national level, for example, as a result of a different uptake of the intervention by different professions. Finally, overhead costs (e.g., administrative, transaction, or building maintenance costs) are not included in the current cost assessment, as they could not be disentangled from the research-related costs. Future research may benefit from a thorough assessment of overhead costs. 

## 5. Conclusions

To conclude, staff costs are an important component of the costs of an alcohol measurement programme, being largely driven by the type and proportion of the professions of providers delivering the intervention and the time spent on the sessions. Regarding the costs of implementation strategies, the standard training and clinical package is the cheapest of the three analysed implementation strategies and may lead, in all three countries, to additional patients whose alcohol consumption is measured, compared to care as usual. On the other hand, more complex and expensive implementation strategies, such as the intensive training and clinical package, or the provision of community support (along with the training and clinical package) may also potentially result in additional alcohol measurement sessions; however, this depends on the country where the strategy is implemented. Therefore, for optimal budgeting efforts of alcohol measurement programmes, increased attention should be paid to the local characteristics of the contexts where the intervention is implemented, thereby contributing to generating evidence for decision making in public health policies.

## Figures and Tables

**Table 1:** Costs units, quantities, and prices of brief alcohol advice sessions.

Unit	Operationalisation	Quantity			Unit Cost			Costs (Int$)		
		Col	Mex	Per	Col	Mex	Per	Col	Mex	Per
Alcohol measurement session	Minutes spent by provider to measure alcohol use in a new patient, using the AUDIT-C questionnaire.	4.3 (CI:3.46; 5.13)	2.43 (CI:0.75; 4.1)	4.73 (CI:4.54; 4.93)	Int$ 15.69 per hour	Int$ 13.77 per hour	Int$ 12.76 per hour	1.12 (CI:0.90; 1.34)	0.57 (CI:0.17; 0.94)	1.01 (CI:0.97; 1.05)
Brief advice session	Minutes spent by provider to deliver a brief advice session to a patient.	5.26 (CI:4.27; 6.25)	4.14 (CI:1.35; 6.94)	4.85 (CI:4.59; 5.12)				1.38 (CI:1.12; 1.63)	0.95 (CI:0.31; 1.59)	1.03 (CI:0.98; 1.09)
Referral to treatment session	Minutes spent by provider to deliver a referral to treatment session to a patient.	2.50 (CI:1.94; 3.06)	2.43 (CI:0.63; 4.22)	1.60 (CI:1.46; 1.74)				0.65 (CI:0.54; 0.80)	0.56 (CI:0.14; 0.97)	0.34 (CI:0.31; 0.37)
Alcohol measurement material	Number of double-sided pages used for the AUDIT-C tally sheet, for each new patient whose alcohol consumption is measured.	1	1	1	Int$ 0.07 per page	Int$ 0.1 per page	Int$ 0.1 per page	0.07	0.10	0.10
Consultation cost and alcohol measurement session	Costs incurred for every new patient whose alcohol consumption was measured, who did not receive subsequent interventions (staff costs + materials).							1.19 (CI:0.97; 2.54)	0.67 (CI:0.27; 1.04)	1.11 (CI:1.07; 1.15)
Consultation cost, alcohol measurement, and brief advice session	Costs incurred for every new patient whose alcohol consumption was measured and received brief advice (staff costs + materials).							2.57 (CI:2.09; 4.17)	1.62 (CI:0.58; 2.63)	2.14 (CI:2.05; 2.24)
Consultation cost, alcohol measurement, and referral to treatment session	Costs incurred for every new patient whose alcohol consumption was measured and received referral to treatment (staff costs + materials).							1.84 (CI:1.51; 3.34)	1.23 (CI:0.41; 2.01)	1.45 (CI:1.38; 1.52)

**Table 2:** Estimated costs per 10,000 patients whose alcohol consumption would be measured in one study arm.

	Nr. of Alcohol Measurement Sessions Delivered in 5 Months of SCALA Implementation, and nr. of Participating Providers and PHCCs, per Study Arm.			Period within Which 10,000 Alcohol Measurement Sessions Would Be Delivered in One SCALA Study Arm			Programme and Consultation Costs for 10,000 Alcohol Measurement Sessions (Int$)		
	Col	Mex	Per	Col	Mex	Per	Col	Mex	Per
Standard training and clinical package	446 (30 providers in five PHCCs)	590 (54 providers in five PHCCs)	846 (70 providers in five PHCCs)	9.34 years	7.06 years	4.92 years	20,082.85	22,177.18	25,474.28
Community support (in SCALA combined with standard training and clinical package)	1830 (26 providers in five PHCCs)	922 (59 providers in five PHCCs)	566 (40 providers in five PHCCs)	2.27 years	4.51 years	7.36 years	24,654.26	27,474.40	34,103.66
Intensive training and clinical package (in SCALA combined with community support)	1222 (17 providers in five PHCCs)	1313 (47 providers in four PHCCs)	258 (50 providers in five PHCCs)	3.40 years	3.17 years	16.14 years	37,506.26	30,360.53	74,414.28

## Data Availability

According to the project’s data management plan, all quantitative datasets generated in the course of the SCALA study will be made openly available.

## Appendix A

**Table A1:** Description of the health care systems and participating municipalities in the three countries.

	Colombia	Mexico	Peru
Health care systems	In Colombia, the health care system is regulated by the General System of Social Security in Health, which entails an universal health insurance programme managed by insurance companies. By 2017, 95% of the population were covered by public health insurance. Approximately 10% of the population (mainly in the higher SES segment) also own a private insurance scheme.	In Mexico, the health care system is segmented into several partially overlapping groups: (1) social security, covering workers and their families (about 63%), (2) “Seguro Popular”—a public health insurance conceived to provide health care for everyone (poor persons are exempted from paying insurance fees, about 46%), (3) private insurance (about 10%).	In Peru, the health care system is organised into five main programmes: (1) the “Seguro Integral de Salud/SIS“ programme, regulated by the Ministry of Health (for about 47% of the population); (2) the “EsSalud“ programme—a standard contributory health insurance scheme regulated by the Ministry of Labour and Employment Promotion, providing mandatory coverage for formal workers (25% of the population); (3) a programme regulated by the Armed Forces; (4) a programme regulated by the National Police; and (5) the private sector. The latter three sectors are estimated to cover health care services of about 5% of the population
Municipalities	With community support: Soacha (~700,000 inhabitants and an area of 184 km^2^, located in the metropolitan area of Bogota, in the department of Cundinamarca) Without community support: Funza (population ~75,000) and Madrid (population ~80,000) are located at 25 km distance from Bogota, in the department of Cundinamarca.	With community support: three municipalities in Mexico City: Tlaplan (~650,000 inhabitants); Benito Juares (~400,000 inhabitants); and Alvaro Obregon (~700,000 inhabitants) Without community support: two municipalities in Mexico City: Miguel Hidalgo (~400,000 inhabitants) and Xochimilco (~400,000 inhabitants)	With community support: Callao (~450,000 inhabitants, xx km), located in the proximity of Lima and of the Pacific Ocean, in the province Callao. Without community support: two districts in the Lima region: Chorrilos (~300,000 inhabitants, xx km) and Santiago de Surco (~300,000 inhabitants, xx km).
	Infrastructure: congested traffic; private transportation services (e.g., taxi, hired minibuses) had to be used for project activities, rather than public transportation. Internet penetrance: medium, ~60% of the population has access to internet.	Infrastructure: congested traffic; private transportation services (e.g., taxi, hired minibuses) had to be used for project activities, rather than public transportation. Internet penetrance: medium, ~70% of the population has access to internet.	Infrastructure: congested traffic and safety risks in certain regions; private transportation services (e.g., taxi, hired minibuses) had to be used for project activities, rather than public transportation. Internet penetrance: medium, ~60% of the population has access to internet.

**Table A2:** Alcohol measurement-related activities and materials offered to participants in different study arms.

SCALA Study Arms			
Arm 1 Care as Usual	Arm 2 Standard Training and Clinical Package	Arm 3 Community Support	Arm 4 Intensive Training and Clinical Package
Participants received: - A booklet describing a pathway for delivering alcohol measurement and subsequent interventions; - paper tally sheets with the AUDIT-C questionnaire (3 items) which could be used by providers to deliver the intervention; - no other support materials and activities were offered.	Participants received: - A booklet describing a pathway for delivering alcohol measurement and subsequent interventions; - paper tally sheets with the AUDIT-C questionnaire (3 items) which could be used by providers to deliver the intervention; - training (one session) before the start of the implementation, in which they were trained to deliver alcohol measurement and subsequent interventions using the same pathway as in arm 1; - patient leaflets (2 double-sided pages) to be offered to patients receiving brief advice.	Participants received: - A booklet describing a pathway for delivering alcohol measurement and subsequent interventions; paper tally sheets with the AUDIT-C questionnaire (3 items) which could be used by providers to deliver the intervention; - training (one session) before the start of the implementation, in which they were trained to deliver alcohol measurement and subsequent interventions using the same pathway as in arm 1; - patient leaflets (2 double-sided pages) to be offered to patients receiving brief advice; community support was offered to participating PHCC, in the form of supportive actions (e.g., regular feedback offered to providers) and CAB meetings.	Participants received: - A booklet describing a pathway for delivering alcohol measurement and subsequent interventions; paper tally sheets with the AUDIT questionnaire (10 items) which could be used by providers to deliver the intervention; - one training (two sessions in Mexico and Peru, one session in Colombia, which was longer that in the other arms) before the start of the implementation, in which they were trained to deliver alcohol measurement and subsequent interventions using a longer pathway than in arm 1; - patient leaflets (2 double-sided pages) to be offered to patients receiving brief advice; community support was offered to participating PHCC, in the form of supportive actions (e.g., regular feedback offered to providers) and CAB meetings.

**Table A3:** SCALA community support implemented in the three intervention municipalities until end of implementation, month 5.

Community Support Activity	Colombia	Mexico	Peru
CAB meetings	Two CAB meetings setting up the Municipal Actions Plan for the community actions intervention.	Two CAB meetings setting up the Municipal Actions Plan for the community actions intervention.	One CAB meeting setting up the Municipal Actions Plan for the community actions intervention.
Adoption mechanisms	1. The benefits of the SCALA project have been emphasised in face-to-face meetings with centre managers and providers. 2. In implementation month 3, in a face to face meetings with providers, the number of screened patients was communicated. 3. A local university became engaged in the project. 4. In implementation month 3, in face to face meetings with providers, the highest alcohol measurement rates per centre were highlighted. 5. Organisational issues are monitored through discussions with centres; no substantial issues have been identified.	1. During the training sessions, the benefits of implementing the alcohol measurement and brief advice in the centre for patients, providers, and the community have been highlighted. 2. In the training sessions, the large number of patients that can benefit if alcohol measurement and brief advice are implemented in the centre was reaffirmed. 3. A poster presentation was held at an Annual Research Meeting of the National Institute of Psychiatry; a presentation about the role of alcohol measurement was held on the National Day against Harmful use of Alcoholic Beverages 2019. 4. Informing centres about the percentage of alcohol measurement sessions carried out by each centre is done on a monthly basis. 5. Organisational issues are monitored through discussions with centres; no substantial issues have been identified.	1. Collaboration with the Mental Health Program of the Ministry of Health, in order to promote the adoption of the program in the implementation municipality. 2. The large number of patients who benefit from the project was communicated to providers, focusing on three subgroups with higher alcohol risk in the intervention municipality: (a) persons in treatment of tuberculosis, (b) persons at risk of sexual transmitted diseases, and (c) persons in violent families. 3. In order to engage the municipality, 35 community promoters were trained in methods for working in alcohol prevention. 4. Lists were created for each centre using WhatsApp to promote the identification of champions. 5. Organisational issues are monitored through discussions with centres; one issue identified is that providers seem very busy.
Support systems	1. Training packages were slightly shortened, in order to fit into the centres’ schedules and rules of attendance of providers. 2. One formal meeting was organised in the first two months of implementation to identify difficulties regarding the brief advice and the care pathway. 3. Meetings for feedback with providers held every two months, in which the alcohol measurement rates are communicated.	1. Materials and activities of the training sessions were adjusted to the needs of each centre. 2. Reporting the number of alcohol measurement sessions to centres each month; informing centres every three months on the progress of the global project.	1. Additional materials were added for new providers who did not have previous information about the program. 2. Reporting the number of alcohol measurement sessions each month to centres. 3. Exploring the option of involving Community Mental Health Services, who could train other centres in the future.

**Table A4:** Average hourly salary of different health care professionals involved in the SCALA project (Int$).

Profession	Colombia	Mexico	Peru
GP	20.43	16.92	21.53
Nurse	8.18	10.07	9.83
Social worker	11.00	9.49	11.66
Psychologist	14.95	11.41	12.02
Other	11.87	6.41	14.13

**Table A5:** Period within which 10,000 alcohol measurement sessions would be delivered in one SCALA study arm and corresponding number of implementation activities.

	Colombia	Mexico	Peru
Standard training and clinical package	9.34 years Activities: - Start-up for five PHCCs and adaptation of clinical package materials. - One training in the first implementation year for 13 trained providers. - Eight boosters (one per following implementation year) for 13 trained providers.	7.06 years Activities: - Start-up for five PHCCs and adaptation of clinical package materials. - Three trainings in the first implementation year for 44 trained providers. - Eighteen boosters (one per following implementation year) for 44 trained providers.	4.92 years Activities: - Start-up for five PHCCs and adaptation of clinical package materials. - Four trainings in the first implementation year for 61 trained providers. - Twelve boosters (one per following implementation year) for 61 trained providers.
Community support (in SCALA combined with standard training and clinical package)	2.27 years Activities: - Start-up for five PHCCs and adaptation of clinical package materials. - Two trainings in the first implementation year for 21 trained providers. - Two boosters (one per following implementation year) for 21 trained providers. - Three CAB meetings (two in the first year and one per following implementation year). - Set-up and implementation of supportive actions for 5 PHCCs.	4.51 years Activities: - Start-up for five PHCCs and adaptation of clinical package materials. - Three trainings in the first implementation year for 45 trained providers. - Nine boosters (one per following implementation year) for 45 trained providers. - Five CAB meetings (two in the first year and one per following implementation year). - Set-up and implementation of supportive actions for 5 PHCCs.	7.36 years Activities: - Start-up for five PHCCs and adaptation of clinical package materials. - Two trainings in the first implementation year for 34 trained providers. - Twelve boosters (one per following implementation year) for 34 trained providers. - Seven CAB meetings (one per implementation year). - Set-up and implementation of supportive actions for 5 PHCCs.
Intensive training and clinical package (in SCALA combined with community support)	3.40 years Activities: - Start-up for five PHCCs and adaptation of clinical package materials. - One intensive training (one session) in the first implementation year for 17 trained providers. - Two boosters (one per following implementation year) for 15 trained providers. - Fours CAB meetings (two in the first year and one per following implementation year). -Set-up and implementation of supportive actions for 5 PHCCs.	3.17 years Activities: - Start-up for four PHCCs and adaptation of clinical package materials. - Three intensive trainings (two sessions each) in the first implementation year for 50 trained providers. - Six boosters (one per following implementation year) for 38 trained providers. - Fours CAB meetings (two in the first year and one per following implementation year). - Set-up and implementation of supportive actions for 5 PHCCs.	16.14 years Activities: - Start-up for five PHCCs and adaptation of clinical package materials. - Three intensive trainings (two sessions each) in the first implementation year for 41 trained providers. - Forty-five boosters (one per following implementation year) for 41 trained providers. - Fours CAB meetings (two in the first year and one per following implementation year). - Set-up (40 h) and implementation of supportive actions for 5 PHCCs (15 h monthly).

**Table A6:** Units, quantities, and costs of implementation strategies.

Unit	Unit Operationalisation	Quantity			Unit Cost (Int$)			Costs (Int$)		
		Col	Mex	Per	Col	Mex	Per	Col	Mex	Per
Set-up and Adaptation Costs										
Coordination of PHCC participation	Hours spent to coordinate participation of one PHCC.	10 h	10 h	10 h	14.95 per hour	11.41 per hour	12.02 per hour	149.50	114.08	120.21
Coordination user panel	Hours spent to coordinate and organise one user panel.	20 h	20 h	20 h	14.95 per hour	11.41 per hour	12.02 per hour	299.00	228.17	40.41
Food and refreshments	Food and refreshments in one user panel with 10 participants, including moderator and organiser.	12 portions	12 portions	12 portions	2.59	4.51	6.89	31.13	54.14	82.66
Materials	Number of materials used during one user panel with 10 participants.	10 sets	10 sets	10 sets	1.48 per set	3.65 per set	3.10 per set	14.83	36.52	31.00
Remuneration moderator user panel	Number of hours spent by the moderator to prepare and deliver one user panel with 10 participants.	4 h	4 h	4 h	14.95 per hour	11.41 per hour	12.02 per hour	59.80	45.63	48.08
Transportation	Transportation used for one user panel with 10 participants, including moderator and organisers.	One transporation service	One transporation service	One transporation service	37.06	107.42	71.76	37.06	107.42	71.76
Adaptation of materials based on feedback	Hours spent to implement adaptation and tailoring of the clinical package materials.	30 h	30 h	30 h	14.95 per hour	11.41 per hour	12.02 per hour	448.50	342.25	360.62
Total set-up costs	Costs for coordinating the participation of 15 PHCCs in arms 2, 3, and 4.							2242.50	1711.25	1803.10
Total costs adaptation materials	Costs for two user panels and further adaptation of the clinical package materials.							1332.15	1286.02	1308.45
Standard Training and Clinical Package										
Training coordination	Number of hours spent to coordinate one training session with 15 participants.	20 h	20 h	20 h	14.95 per hour	11.41 per hour	12.02 per hour	299.00	228.17	240.41
Participants materials	Number of materials used during one training session with 15 participants.	15 sets	15 sets	15 sets	2.59 per set	5.37 per set	5.74 per set	38.92	80.57	86.11
Remuneration trainer	Number of hours spent by the trainer to prepare and deliver one training session with 15 participants.	3 h	4 h	4 h	18.82 per hour	20.41 per hour	16.19 per hour	56.46	81.64	64.75
Food and refreshments (one training)	Food and refreshments in one training session with 15 participants, including trainer and organiser.	17 portions	17 portions	17 portions	2.59 per portion	4.51 per portion	6.89 per portion	44.11	76.70	117.11
Transportation (one training)	Transportation used for one training session with 15 participants, including trainer and organisers.	One transporation service	One transporation service	One transporation service	37.06	75.20	71.76	37.06	75.20	71.76
Total costs for one standard training								475.55	542.27	580.14
Total costs for one trained provider (standard training)								31.70	36.15	38.68
Clinical package materials for alcohol measurement	Number of double-sided pages used in the standard clinical package for each new patient whose alcohol consumption is measured.	2 double- sided pages	2 double- sided pages	2 double- sided pages	0.15 per double- sided page	0.27 per double- sided page	0.26 per double- sided page	0.30	0.54	0.52
Intensive Training and Clinical Package										
Total costs for one intensive training	Total costs for one intensive training consisting of one session in Colombia and two sessions in Mexico and Peru.							547.09	1050.22	881.99
Total costs for one trained provider (intensive training)								36.47	63.01	64.14
Additional costs intensive training per provider (compared to standard training)	Additional costs, per provider, spent to provide intensive training (over and above standard training).							6.50	30.01	21.11
Additional time full AUDIT	Additional number of minutes spent to measure the alcohol consumption of a new patient, with the full AUDIT (over and above AUDIT-C).	3.75 min	2 min	5 min	15.69 per hour	13.77 per hour	12.76 per hour	0.98	0.46	1.06
Additional alcohol measurement material	Number of additional double-sided pages used for the full AUDIT assessment, for each new patient whose alcohol consumption is measured (as compared to care as usual).	1	1	1	0.07 per page	0.1 per page	0.1 per page	0.07	0.10	0.10
Community Support										
CAB coordination and moderation	Number of hours spent to prepare and coordinate one CAB meeting.	35 h	35 h	35 h	14.95 per hour	11.41 per hour	12.02 per hour	523.25	399.29	420.72
Venue rent	Venue rent for one CAB meeting.	1 conference hall	1 conference hall	none	111.19	268.56	--	111.19	268.56	0.00
Food and refreshments	Food and refreshments used in one CAB meeting.	12 portions	12 portions	12 portions	2.59 per portion	4.51 per portion	9.57 per portion	31.13	54.14	82.66
Materials	Amount of materials used in one CAB meeting.	10 sets	10 sets	10 sets	1.48 per set	3.65 per set	3.05 per set	14.80	32.52	30.54
Transportation	Transportation used for one CAB meeting.	One transporation service	One transporation service	One transporation service				37.06	75.20	71.76
Total cost one CAB meeting	Total costs for one CAB meeting.							717.44	833.71	605.68
Set-up supportive actions	Amount of hours spent to set-up and prepare supportive actions for 1 municipality, including 10 PHCCs.	40 h	40 h	40 h	14.95 per hour	11.41 per hour	12.02 per hour	598.00	456.33	480.83
Coordination and implementation supportive actions	Amount of hours spent to implement supportive actions for one municipality, including 10 PHCCs, during 1 month.	20 h	10 h	10 h	14.95 per hour	11.41 per hour	12.02 per hour	299.00	171.12	120.21
Total cost of supportive actions (1 municipality, 5 months)	Total costs of implementing supportive actions in 1 municipality, including 10 PHCCs.							1495.00	855.62	601.03
Total costs of community support	Total costs of 5 months of community support in 1 municipality, consisting of two CAB meetings in Colombia and Mexico, one CAB meeting in Peru, and five months of supportive actions.							2929.88	2523.04	1206.72
